# Activity of Drug Combinations against *Mycobacterium abscessus* Grown in Aerobic and Hypoxic Conditions

**DOI:** 10.3390/microorganisms10071421

**Published:** 2022-07-14

**Authors:** Alessio Lanni, Emanuele Borroni, Angelo Iacobino, Cristina Russo, Leonarda Gentile, Lanfranco Fattorini, Federico Giannoni

**Affiliations:** 1 Department of Infectious Diseases, Istituto Superiore di Sanità, 00161 Rome, Italy; alessio.lanni@guest.iss.it (A.L.); angelo.iacobino@iss.it (A.I.); lanfranco.fattorini@iss.it (L.F.); 2 Emerging Bacterial Pathogens Unit, San Raffaele Scientific Institute, 20132 Milan, Italy; borroni.emanuele@hsr.it; 3 Bambino Gesù Children’s Hospital, 00165 Rome, Italy; cristina.russo@opbg.net (C.R.); leonarda.gentile@opbg.net (L.G.)

**Keywords:** *Mycobacterium abscessus*, cystic fibrosis, aerobiosis, anaerobiosis, nitrocompounds, colistin, nimorazole, persisters, drug combinations, drug tolerance

## Abstract

Infections caused by *Mycobacterium abscessus* (Mab), an environmental non-tuberculous mycobacterium, are difficult to eradicate from patients with pulmonary diseases such as cystic fibrosis and bronchiectasis even after years of antibiotic treatments. In these people, the low oxygen pressure in mucus and biofilm may restrict Mab growth from actively replicating aerobic (A) to non-replicating hypoxic (H) stages, which are known to be extremely drug-tolerant. After the exposure of Mab A and H cells to drugs, killing was monitored by measuring colony-forming units (CFU) and regrowth in liquid medium (MGIT 960) of 1-day-old A cells (A1) and 5-day-old H cells (H5). Mab killing was defined as a lack of regrowth of drug-exposed cells in MGIT tubes after >50 days of incubation. Out of 18 drugs tested, 14-day treatments with bedaquiline-amikacin (BDQ-AMK)-containing three-drug combinations were very active against A1 + H5 cells. However, drug-tolerant cells (persisters) were not killed, as shown by CFU curves with typical bimodal trends. Instead, 56-day treatments with the nitrocompounds containing combinations BDQ-AMK-rifabutin-clarithromycin-nimorazole and BDQ-AMK-rifabutin-clarithromycin-metronidazole-colistin killed all A1 + H5 Mab cells in 42 and 56 days, respectively, as shown by lack of regrowth in agar and MGIT medium. Overall, these data indicated that Mab persisters may be killed by appropriate drug combinations.

## 1. Introduction

Non-tuberculous mycobacteria (NTM) are environmental organisms increasingly recognized as human pathogens [1]. Among the rapidly growing NTM, the *Mycobacterium abscessus* (Mab) complex includes the subspecies *massiliense*, *bolletii* and *abscessus* [2]. *M. abscessus* is becoming the most prominent and worrisome NTM pathogen worldwide for its capacity to cause chronic infections in patients with pre-existing pulmonary lesions, including people with cystic fibrosis and bronchiectasis [2,3,4]. 

*M. abscessus* infections in patients with cystic fibrosis are increasing [5,6]. Cystic fibrosis arises due to mutational dysfunctions of the gene encoding the transmembrane conductance regulator protein [7]. When this protein is not functional, thick airway secretions impair mucociliary lung clearance, which increases bacterial colonization. Organisms inside cystic fibrosis lungs form biofilms within the thickened alveolar walls and airways [1,8]. When biofilms reach a thickness of >40 nm, oxygen depletion occurs [9], and Mab may restrict growth from actively replicating (AR) aerobic to non-replicating (NR), anaerobic, drug-tolerant cells (persisters). An extreme drug tolerance to Mab persisters was reported [10]. As seen for *Mycobacterium tuberculosis* (Mtb), NTM may enter an NR state, exhibiting high phenotypic drug resistance inside lung lesions [1]. Once the infection is established, Mab colonial morphology may also change from the smooth to the rough variant, which is associated with increased antibiotic and hydrogen peroxide resistance and with invasive behaviour [6,11]. 

*M. abscessus* pulmonary diseases are treated with antibiotic combinations for months or years, but often eradication fails and leads to an accelerated decline in lung functions [2,12]. In the US and Europe, recommendations for the management of NTM in cystic fibrosis [13] with Mab treatment include an initial intensive phase for 3–12 weeks with a macrolide and intravenous amikacin (AMK), with one or more additional drugs, including intravenous tigecycline (TGC), imipenem or cefoxitin, followed by a continuation phase of >1 year of a macrolide and inhaled AMK with two to three additional agents, including clofazimine (CLF), linezolid (LNZ), moxifloxacin (MXF) or minocycline. Recent studies showed promising activity against Mab by bedaquiline (BDQ), rifabutin (RFB) and other drugs [14,15,16,17]. A recent paper reported that the combination BDQ-AMK-RFB was bactericidal against AR and nutrient-starved Mab [18], but no information was shown on its activity under hypoxic conditions, which may mimic the environment of the hypoxic biofilm in the alveoli of cystic fibrosis patients better than nutrient starvation.

Previous studies by our group and other investigators reported consistent activities of nitrocompounds [19,20], including metronidazole (MTR), pretomanid (PRT, formerly PA-824) and nitazoxanide (NTZ), alone and in combination, against AR and NR Mtb [21,22,23,24,25,26]. Recent trials demonstrated the potential of PRT-containing regimens against drug-susceptible and -resistant pulmonary tuberculosis (TB) [27,28]. As for Mab, some activity of nitrocompounds such as niclosamide (NCL) was reported against nutrient-starved Mab [29], while no activities of MTR [30] and of PRT against AR Mab [31] were found.

Here, we systematically investigated whether adding nitrocompounds and other agents to drugs currently used in the therapy may improve activity against AR and NR Mab. Overall, these observations allowed us to find two combinations killing both Mab metabolic stages.

## 2. Materials and Methods

### 2.1. Microorganisms

The *M. abscessus* strain 10 (Mab-10) used throughout this study was obtained from the European Centre for Disease Prevention and Control and identified as *M. abscessus abscessus* by the line probe assay GenoType NTM-DR (Hain Lifescience, Nehren, Germany). Mab-10 showed rough colonies on Middlebrook 7H10 agar (Difco, Detroit, MI, USA) supplemented with 10% oleic acid-albumin-dextrose-catalase (OADC) (Becton Dickinson, Sparks, MD, USA). The strain was a clinical isolate from a lung infection.

The minimum inhibitory concentrations (MICs) of Mab-10 were determined by the broth microdilution method using the Sensititre RAPMYCOI plates (Thermo Fisher Scientific, Waltham, MA, USA). MICs were recorded after 5 days of incubation, with the exception of those of clarithromycin (CLR), which were recorded on days 5 and 14 in order to assess inducible CLR resistance [32]. The MICs of BDQ, RFB and CLF were performed with the same protocol of the RAPMYCOI assay. All MIC values are shown in the Supplementary Table S1. MIC interpretation was performed according to the Clinical and Laboratory Research Institute (CLSI) breakpoints [32] and showed that Mab-10 was susceptible only to CLR and AMK. These results were confirmed by the Whole Genome Sequence analysis of *erm(41)*, *rrl* and *rrs* genes [5,33,34].

### 2.2. Growth of Mab-10 under Aerobic (A) and Hypoxic (H) Conditions

Mab-10 was grown in 20 by 125 mm screw-cap tubes containing Middlebrook 7H9 broth (Difco) supplemented with 10% Middlebrook albumin-dextrose-catalase (ADC) (Becton Dickinson) and stirred with 8-mm magnetic bars, mainly as described in the Mtb Wayne dormancy culture model [21,22,24,25,35]. The major difference between Mtb and Mab Wayne models is that Mab-10 was grown in 7H9 broth instead of Dubos tween albumin broth (DTA, used for Mtb), because Mab rapidly formed clumps and pellicles on the top surface of DTA culture tubes, making colony forming units (CFU) results unreliable. 

For the preparation of AR aerobic (A) cells, mid-log phase cultures were diluted in 7H9 broth and transferred to tubes in 12 mL volumes. Tubes were incubated at 37 °C with loosened screw caps and stirred at 120 rpm. For the preparation of NR hypoxic (H) cells, mid-log phase cultures were diluted and transferred to tubes in 16 mL volumes, but in this case, to obtain anaerobic conditions, the caps were tightly screwed and tight rubber caps were put under the caps. H tubes were incubated at 37 °C and stirred at 120 rpm. Control H tubes with 1.5 µg/mL of methylene blue as an indicator of oxygen depletion were added in each experiment [35]. Mycobacterial growth was monitored by measuring optical density at 600 nm (OD) and CFU/mL on Middlebrook 7H10 agar plates incubated at 37 °C under 5% CO_2_ for about 1 week. 

### 2.3. Measurement of Drug Activity against A and H Cells 

The activity of 8 anti-Mab drugs, 9 nitro-compounds and colistin (CLS) was tested against A and H cells. The list of drugs is shown in Supplementary Table S2 [21,22,25,36,37,38,39,40,41,42,43,44,45,46,47]. The drugs were purchased from Sigma-Aldrich (St. Louis, MO, USA) or Selleck Chemicals (Houston, TX, USA). All drugs were dissolved in dimethyl sulfoxide, with the exception of AMK and CLS, which were dissolved in distilled water. 

To determine drug activity, 1-day-old aerobic (A1) cells and 5-day-old hypoxic (H5) cells were incubated with single drugs or drug combinations. Drugs (100 µL) were added by micropipette to A1 cultures and by syringe to H5 cultures (Wayne model). All drugs were used at their maximum serum concentration (C_max_), with the exception of RFB, which was used at the C_max_ in the lung tissue [36], and of CLS, which was used at 25 and 100 µg/mL [47].

After incubation, at various times, 1 mL of A1 or H5 cultures was washed and resuspended in 1 mL of 7H9 broth, and 0.2 mL was inoculated in Middlebrook 7H10 agar plates for CFU determination and in liquid medium (Bactec MGIT 960 system; Becton Dickinson) for the determination of the number of days to reach a growth unit of ≥75 (days to positivity (DTP)). Mab killing was defined as a lack of regrowth in MGIT tubes after >50 days of incubation, which is similar to our previous studies on Mtb [21,25].

## 3. Results

### 3.1. Growth of Mab-10 under A and H Conditions

**Figure 1:**
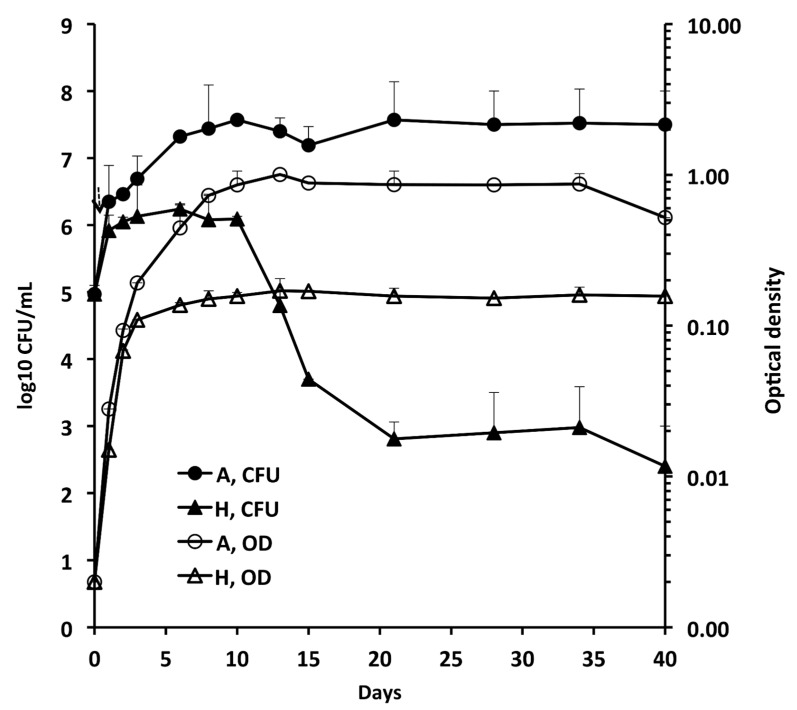
Growth of Mab-10 in aerobic (A), and hypoxic (H) conditions (Wayne dormancy culture model). Means ± standard deviations of colony forming units (CFU)/ml and optical density (OD) values are shown.

Figure 1 shows the growth of A and H cells by measuring CFU and OD for 40 days. The CFU and OD of A cultures increased up to day 10 and then remained quite stable up to day 40. Contrastingly, the OD of H cultures was not paralleled by CFU, which sharply decreased by 3 log_10_ from day 10 to 21, followed by stabilization to about 10^3^ CFU/mL. After adding methylene blue to some H tubes as an oxygen indicator, fading and full decolourization of the dye occurred on days 3 and 5, respectively, indicating that anaerobic conditions developed on day 5. 

### 3.2. Activity of the Combination BDQ-AMK-RFB

**Figure 2:**
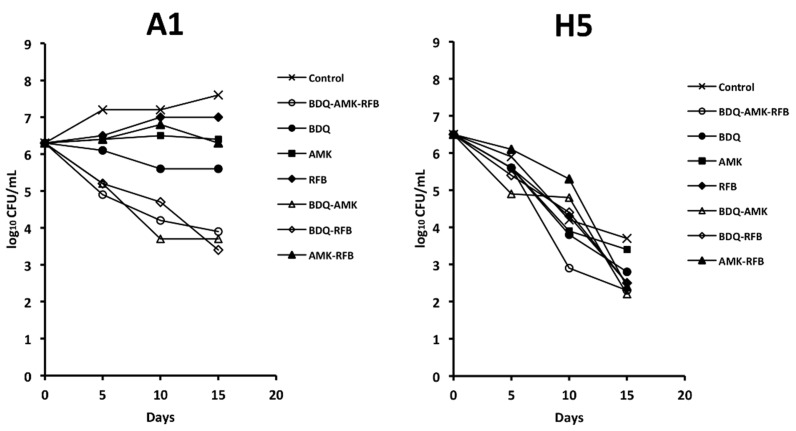
Survival of A1 and H5 cells of Mab-10 after exposure to bedaquiline (BDQ)-amikacin (AMK)-rifabutin (RFB), 2-drug components, and single drugs, as estimated by CFU counts. A representative experiment out of two is shown.

It has been recently reported that the combination BDQ-AMK-RFB was bactericidal against AR and nutrient-starved Mab [18]. Here, we investigated the activity of BDQ-AMK-RFB under hypoxic conditions to mimic low oxygen levels in the mucus and biofilm of the alveoli of cystic fibrosis patients and the lung cavity of individuals with chronic obstructive pulmonary disease [1,8]. Figure 2 shows the activity of BDQ-AMK-RFB and its components against A1 and H5 cells. Bedaquiline inhibited A1 cells to a greater extent than AMK and RFB. BDQ-AMK-RFB, BDQ-RFB and BDQ-AMK were much more potent than single drugs, while low activity was shown for AMK-RFB. These data indicated that BDQ was the pivotal drug of BDQ-AMK-RFB. As for H5 cells, on day 15, the CFU numbers of BDQ-AMK-RFB and its components were usually >1-log lower than untreated control cells, with the activity of BDQ-AMK being similar to that of BDQ-AMK-RFB. The two-drug combination BDQ-AMK was chosen for adding other drugs in order to test new combinations against A1 and H5 cells.

### 3.3. Activity of Three-Drug Combinations Containing BDQ-AMK

**Figure 3:**
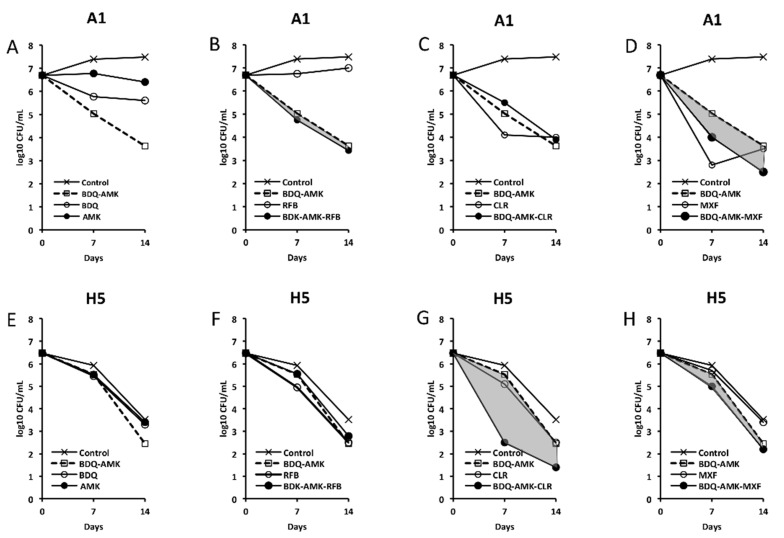
Activity against A1 and H5 cells of 3-drug combinations containing bedaquiline-amikacin (BDQ-AMK). (**A**,**E**) BDQ-AMK and its components BDQ and AMK; (**B**,**F**) BDQ-AMK ± rifabutin (RFB); (**C**,**G**) BDQ-AMK ± clarithromycin (CLR); (**D**,**H**) BDQ-AMK ± moxifloxacin (MXF). Increases in the activities of (BDQ-AMK + third drug) versus BDQ-AMK are highlighted in grey.

**Figure 4:**
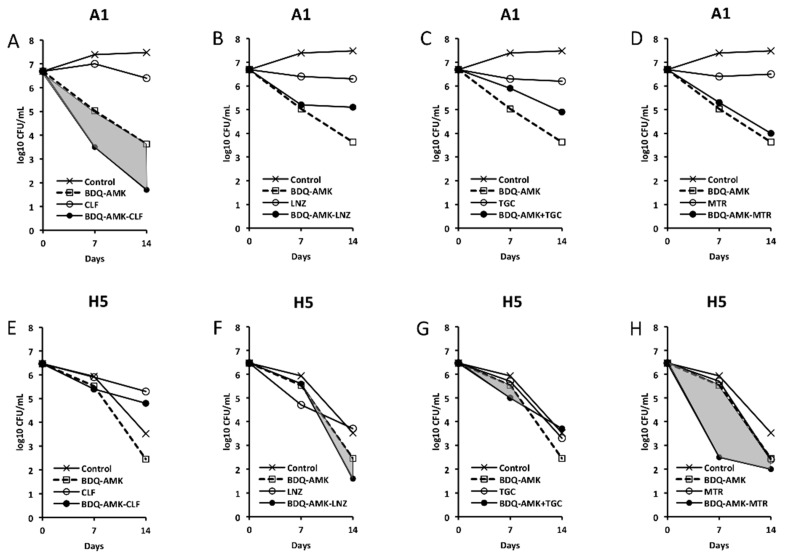
Activity against A1 and H5 cells of 3-drug combinations containing bedaquiline-amikacin (BDQ-AMK). (**A**,**E**) BDQ-AMK ± clofazimine (CLF); (**B**,**F**) BDQ-AMK ± linezolid (LNZ); (**C**,**G**) BDQ-AMK ± tigecycline (TGC); (**D**,**H**) BDQ-AMK ± metronidazole (MTR). Increases in the activities of (BDQ-AMK + third drug) versus BDQ-AMK are highlighted in grey.

**Figure 5:**
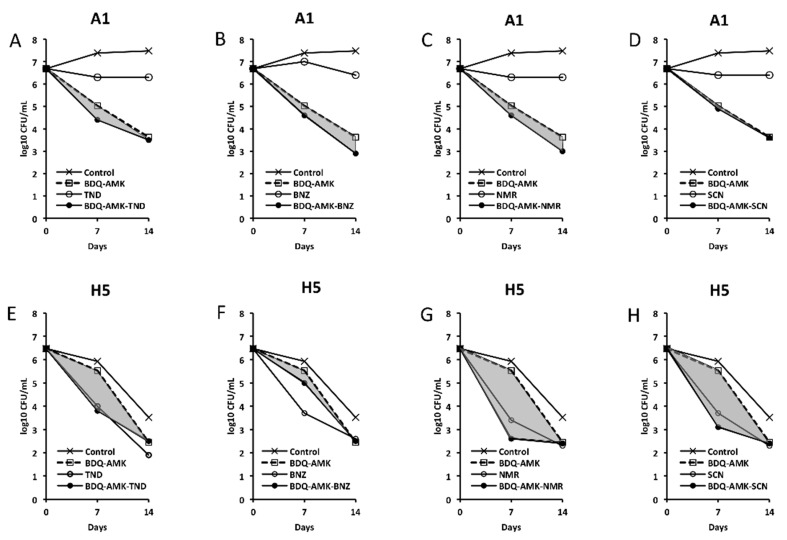
Activity against A1 and H5 cells of 3-drug combinations containing bedaquiline-amikacin (BDQ-AMK). (**A**,**E**) BDQ-AMK ± tinidazole (TND); (**B**,**F**) BDQ-AMK ± benznidazole (BNZ); (**C**,**G**) BDQ-AMK ± nimorazole (NMR); (**D**,**H**) BDQ-AMK ± secnidazole (SCN). Increases in the activities of (BDQ-AMK + third drug) versus BDQ-AMK are highlighted in grey.

**Figure 6:**
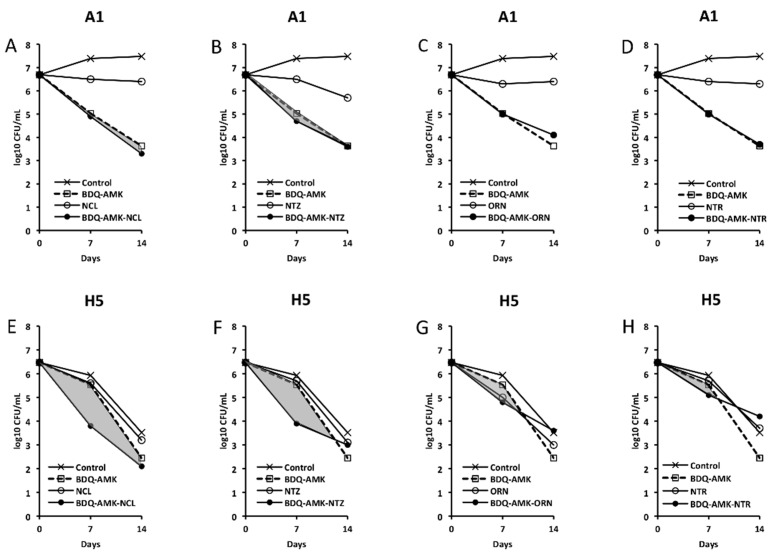
Activity against A1 and H5 cells of 3-drug combinations containing bedaquiline-amikacin (BDQ-AMK). (**A**,**E**) BDQ-AMK ± niclosamide (NCL); (**B**,**F**) BDQ-AMK ± nitazoxanide (NTZ); (**C**,**G**) BDQ-AMK ± ornidazole (ORN); (**D**,**H**) BDQ-AMK ± nitroxoline (NTR). Increases in the activities of (BDQ-AMK + third drug) versus BDQ-AMK are highlighted in grey.

Figure 3, Figure 4, Figure 5 and Figure 6 show the activities of BDQ-AMK plus a third drug. As for the drugs alone, on day 14 the most active agents against A1 cells were MXF and CLR (3.5–4.0 log_10_ CFU reduction, compared to untreated cells) and BDQ (1.9 log_10_ CFU reduction); all other drugs were mostly bacteriostatic. On day 14, the most active agents (alone) against H5 cells were RFB, CLR, MTR, tinidazole (TND), benznidazole (BNZ), nimorazole (NMR) and secnidazole (SCN) (0.9–1.6 log_10_ CFU reduction).

As for combinations, only MXF and CLF increased BDQ-AMK killing (highlighted in grey) of A1 cells on day 7 (1.0–1.5 log_10_ CFU reduction) and 14 (1.1–1.9 log_10_ CFU reduction). Instead, CLR, MTR, TND, NMR, SCN, NCL and NTZ enhanced the BDQ-AMK killing (highlighted in grey) of H5 cells on day 7 (1.6–3.0 log_10_ CFU reduction) but not on day 14, with the exception of CLR (1.0 log_10_ CFU reduction) and MTR (0.5 log_10_ CFU reduction). However, the addition of a third drug did not improve BDQ-AMK killing of both A1 and H5 cells.

### 3.4. Activity of Five-Drug Combinations

**Figure 7:**
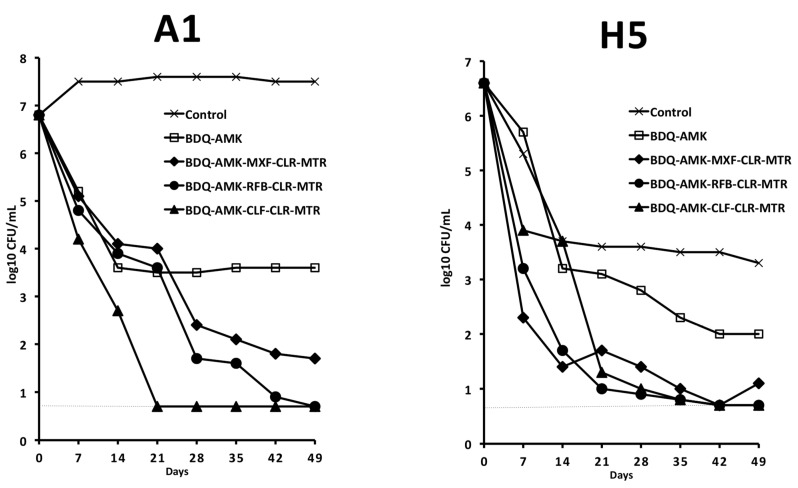
Activity against A1 and H5 cells of 5-drug combinations containing BDQ-AMK added with RFB (or CLF or MXF, both potentiating A1 cells), and with CLR-MTR (potentiating H5 cells). Dashed lines indicate the limit of detection (5 CFU/mL). A representative experiment out of two is shown.

Based on data shown in Figure 3, Figure 4, Figure 5 and Figure 6, long-term experiments were performed to find one or more combinations killing both A1 and H5 cells, as estimated by the lack of regrowth both in solid media (CFU in agar) and liquid media (DTP > 50 days in MGIT 960 tubes). Firstly, we tested combinations containing BDQ-AMK added with RFB (or CLF or MXF, both potentiating A1 killing) and with CLR-MTR (potentiating H5 killing) (Figure 7). BDQ-AMK-CLF-CLR-MTR decreased A1 CFU below the detection limit of the method (5 CFU/mL) in 21 days. BDQ-AMK-RFB-CLR-MTR and BDQ-AMK-CLF-CLR-MTR decreased H5 CFU to <5/mL in 42 days, with the first combination lowering CFU more rapidly than the second. Lower activity was shown by BDQ-AMK-MXF-CLR-MTR. 

**Figure 8:**
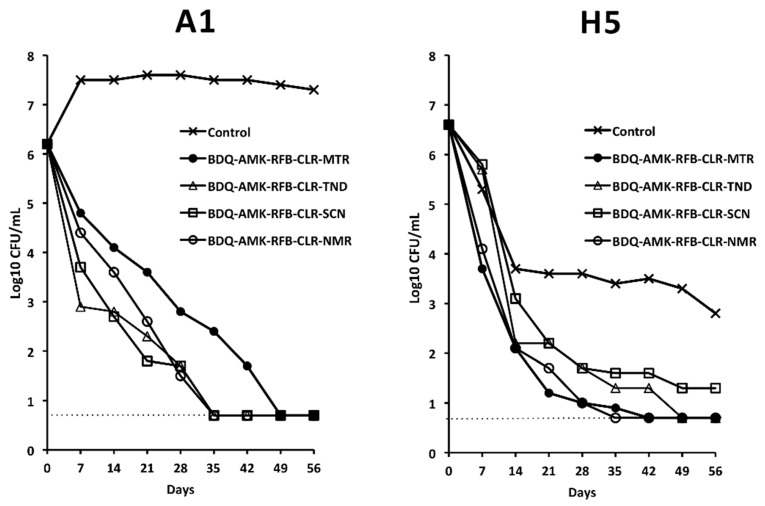
Activity against A1 and H5 cells of BDQ-AMK-RFB-CLR-MTR, without or with substitution of MTR with TND, SCN or NMR. Dashed lines indicate the limit of detection (5 CFU/mL). A representative experiment out of two is shown.

With the aim to primarily kill drug-tolerant H5 cells, we selected BDQ-AMK-RFB-CLR-MTR for further experiments. Furthermore, we substituted the fifth drug (MTR) with a nitrocompound that showed promising activity as a BDQ-AMK enhancer, namely TND, SCN and NMR (Figure 5). Figure 8 showed that, out of the four combinations tested, BDQ-AMK-RFB-CLR-NMR and BDQ-AMK-RFB-CLR-MTR decreased H5 CFU to <5/mL in 35–42 days. BDQ-AMK-RFB-CLR-TND, BDQ-AMK-RFB-CLR-SCN and BDQ-AMK-RFB-CLR-NMR, but not BDQ-AMK-RFB-CLR-MTR, decreased A1 CFU to <5 CFU/mL in 35 days. 

However, to ascertain cell death, aliquots of drug-exposed cells were also inoculated in MGIT 960 liquid medium (Table 1). As shown by the lack of regrowth in MGIT tubes, BDQ-AMK-RFB-CLR-NMR killed A1 and H5 cells in 42 days. BDQ-AMK-RFB-CLR-MTR killed H5 cells in 56 days, while regrowth was seen for A1 cells. BDQ-AMK-RFB-CLR-TND and BDQ-AMK-RFB-CLR-SCN killed A1 cells in 42 days, while regrowth was seen for H5 cells.

### 3.5. Activity of BDQ-AMK-RFB-CLR-MTR plus CLS 

**Figure 9:**
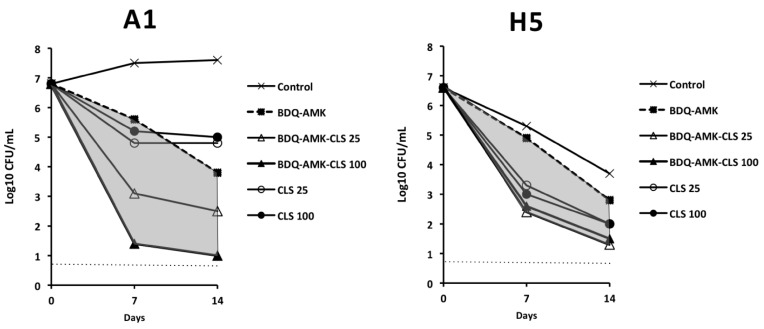
Activity against A1 and H5 cells of 3-drug combinations containing bedaquiline-amikacin (BDQ-AMK) ± 25 µg/mL or 100 µg/mL of colistin (CLS). Increases in the activities of BDQ-AMK-CLS *versus* BDQ-AMK are highlighted in grey. Dashed lines indicate the limit of detection (5 CFU/mL).

Given the difficulty of finding combinations that killed both A1 and H5 cells, we also tested the activity of BDQ-AMK plus CLS. The activity of BDQ-AMK against A1 and H5 cells was greatly enhanced by 25 and 100 µg/mL CLS on day 7 and 14 (Figure 9). 

**Figure 10:**
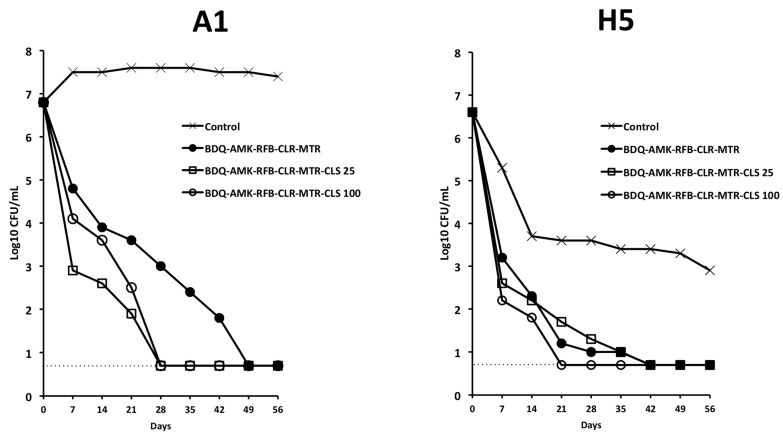
Activity against A1 and H5 cells of BDQ-AMK-RFB-CLR-MTR ±25 µg/mL or 100 µg/mL of CLS. Dashed lines indicate the limit of detection (5 CFU/mL). A representative experiment out of two is shown.

Furthermore, BDQ-AMK-RFB-CLR-MTR-CLS_100_ decreased A1 and H5 CFU more efficiently than BDQ-AMK-RFB-CLR-MTR (Figure 10) and, unlike BDQ-AMK-RFB-CLR-MTR, killed both A1 and H5 cells, as shown by the lack of regrowth in MGIT of A1 cells in 42 days and of H5 cells in 56 days (Table 2). Lower activity against A1 and H5 cells was shown by BDQ-AMK-RFB-CLR-MTR-CLS_25_.

## 4. Discussion

*M. abscessus* is a multidrug-resistant pathogen that has emerged as a global threat in people affected by chronic lung diseases [1,2,3,4]. The organism is an obligate aerobe, but anaerobic conditions like those met in the mucus and biofilm of patients with cystic fibrosis induce the formation of extremely drug-tolerant persisters [10]. In this study, we showed that the patterns of growth of Mab-10 A and H cells were different, with a sharp decrease in H CFU after 10 days and the survival of persisters containing 10^2^–10^3^ CFU/mL.

Firstly, we examined whether the combination BDQ-AMK-RFB, recently described as being active against AR and nutrient-starved Mab [18], was also active in hypoxia. Overall, our data showed that BDQ-AMK-RFB had low levels of activity against H5 cells, consistent with the persistence of drug-tolerant anaerobic Mab in patients, which are indeed rarely cured by antibiotics, even after years of treatment [10]. Due to the comparable activity of BDQ-AMK and BDQ-AMK-RFB against A1 and H5 cells, we then screened three-drug combinations containing BDQ-AMK plus CLR, CLF, RFB, MXF, LNZ, TGC, nitrocompounds, or CLS. The killing of A1 cells by BDQ-AMK was enhanced by CLF, MXF and CLS. As for CLF, reactive oxygen species (ROS)-based killing of Mab [5] and Mtb [48] were reported. As for MFX, a DNA gyrase inhibitor of Mab and Mtb [5], BDQ-MXF-containing combinations showed to be promising regimens against human TB [27]. The killing of H5 cells was enhanced by CLR, nitrocompounds and CLS. Clarithromycin was active both alone and by enhancing BDQ-AMK killing, in keeping with CLR activity reported against biofilm-growing Mab [10]. 

The bimodal killing curves shown by H5 cells treated with BDQ-AMK plus, in particular, CLR, MTR, NMR, SCN or CLS are typical of bacterial persistence. Indeed, the CFU curves were characterized by the first part with the steepest slope (up to day 7), in which the sensitive cells were killed, and a second part, which was flat or with a reduced slope of 1.4–2.4 log_10_ CFU/mL of drug-tolerant surviving cells on day 14 [49]. Bimodal curves were also formed by A1 cells treated with BDQ-AMK plus, in particular, MXF, CLF and CLS. Persisters are likely formed in the hypoxic mucus and biofilm in the alveoli of cystic fibrosis patients, contributing to months/years of drug combination treatments not eradicating them.

Nitrocompounds are known for their ability to generate reactive nitrogen species (RNS) in hypoxic conditions [19,20]. In Mtb, the nitro-imidazole PRT killed NR cells by intracellular nitric oxide release [50], and PRT-containing combinations showed to be promising regimens for the treatment of drug-susceptible and -resistant TB [27,28]. 

Prior to this study, no major information had been reported on the anti-Mab activity of nitrocompounds and CLS. Here, we found that MTR, TND, SCN, NMR and CLS were active against H5 cells both alone and by enhancing BDQ-AMK killing. Furthermore, we found that a five-drug combination containing NMR (BDQ-AMK-RFB-CLR-NMR) killed (DTP ≥ 50 days) A1 + H5 cells in 42 days. The 5-nitroimidazole NMR is active against amoebiasis [51] and trichomoniasis [52], but it is also used as a radiosensitizer to treat head and neck squamous cell carcinomas for sensitizing hypoxic cells to the lethal effects of ionising radiation due to its high electron affinity [53]. At the minimum dose of 1.5 g NMR/day, 5 days a week for 6–7 weeks (C_max_ 36.8 ± 1.3 µg/mL), the side-effects profile of this drug was acceptable, with no lasting toxicity. At higher clinically tolerable doses, C_max_ of 50 µg/mL was reached [53]. Our data showed that BDQ-AMK-RFB-CLR-NMR killed A1 + H5 cells in 42 days; therefore, 6 weeks of treatment with this combination could potentially eradicate Mab. The observation that, among the 5-nitro-imidazoles tested, NMR was the best to enhance A1 + H5 killing, may be tentatively explained with the knowledge that, in hypoxia, the reduction in the nitrogroup produces a nitroradical ion (attacking H5 cells), while, under aerobic conditions, the nitro-radical ion is re-oxidized to form a reactive superoxide anion (attacking A1 cells) [19,20]. This process is known as a “futile cycle”, but, of course, specific studies on the issue are needed.

In the continuous search for new Mab-killing combinations, we also found that BDQ-AMK-RFB-CLR-MTR-CLS_100_ killed A1 + H5 cells in 56 days. Colistin is a membrane-targeting cation polypeptide inhaled two/three times daily in cystic fibrosis patients infected with *Pseudomonas aeruginosa* [54,55]. The drug is nebulized as colistimethate sodium and is found up to ≥128 µg/mL levels in sputum [47]. Colistin is a potentiator of anti-TB drugs [56] and acts by disrupting the Mtb cell wall [57]. In a recent study on new anti-persisters antibiotics, it was found that the lowest numbers of persisters were observed in bacteria treated with molecules targeting the cell membrane, and that CLS was the most effective out of 54 drugs examined [58]. Colistin-treated *Acinetobacter baumannii* cells formed high levels of ROS, supporting the significance of these molecules in decreasing persistence [59]. A ROS-mediated death could also explain our present observations that A1 (but not H5) cells were efficiently killed in a dose-response fashion after the addition of CLS to BDQ-AMK-RFB-CLR-MTR (100 µg/mL were more active than 25 µg/mL CLS).

Overall, in this study, we found two combinations (BDQ-AMK-RFB-CLR-NMR and BDQ-AMK-RFB-CLR-MTR-CLS_100_) killing persister-containing A1 + H5 cells. We are aware that these combinations contain many drugs, but current anti-Mab therapies also involve the administration of several antibiotics for months or years that do not eradicate the organism [10,13]. Thus, the search for new combinations is an urgent necessity. A limitation of this investigation is that our findings on strain Mab-10 may not be necessarily generalized to all Mab isolates, requiring answers in future studies. In this view, we are currently performing studies to find whether these two combinations, or shorter ones, kill A1 + H5 cells generated from Mab isolates in cystic fibrosis. 

In conclusion, the combinations found here showed that it is possible to kill persister-containing Mab cells. Furthermore, we think that our A1 + H5 assay, coupled with a demonstration of the lack of regrowth of drug-treated Mab in solid and liquid media, is a useful tool to find novel anti-mycobacterial combinations.

## Figures and Tables

**Table 1:** Survival of A1 and H5 cells to various drug combinations, as estimated by regrowth in liquid medium using the MGIT 960 system. Day-to-positivity (DTP) values are indicated. Samples inoculated in MGIT 960 tubes refer to samples of Figure 8.

	A1								H5							
	DTP ± SD by Days of Drug Exposure								DTP ± SD by Days of Drug Exposure							
Combination	7	14	21	28	35	42	49	56	7	14	21	28	35	42	49	56
Control	1	1	1	1	1	1	1	1	1	2 ± 1	3 ± 1	3 ± 1	3 ± 1	3 ± 2	3 ± 2	4 ± 1
BDQ-AMK-RFB-CLR-MTR	2	2 ± 1	3 ± 1	3 ± 1	4 ± 1	5 ± 1	7 ± 2	7 ± 3	3 ± 1	5 ± 1	6 ± 1	6 ± 2	6 ± 2	7 ± 3	7 ± 2	>50
BDQ-AMK-RFB-CLR-TND	3 ± 1	3 ± 1	4 ± 1	4 ± 2	20 ± 5	>50	>50	>50	2 ± 1	4 ± 1	4 ± 1	5 ± 1	5 ± 1	6 ± 2	7 ± 2	7 ± 4
BDQ-AMK-RFB-CLR-SCN	2 ± 1	3 ± 1	4 ± 1	6 ± 1	28 ± 4	>50	>50	>50	2 ± 1	3 ± 1	4 ± 1	5 ± 1	5 ± 1	6 ± 1	7 ± 4	7 ± 3
BDQ-AMK-RFB-CLR-NMR	2	4 ± 1	5 ± 1	6 ± 1	25 ± 2	>50	>50	>50	2 ± 1	5 ± 1	5 ± 1	6 ± 1	7 ± 3	>50	>50	>50

**Table 2:** Survival of A1 and H5 cells to various drug combinations, as estimated by regrowth in liquid medium using the MGIT 960 system. Day-to-positivity (DTP) values are indicated. Samples inoculated in MGIT 960 tubes refer to samples of Figure 10.

	A1								H5							
	DTP ± SD by Days of Drug Exposure								DTP ± SD by Days of Drug Exposure							
Combination	7	14	21	28	35	42	49	56	7	14	21	28	35	42	49	56
Control	1	1	1	1	1	1	1	1	1	3 ± 1	3 ± 1	3 ± 1	3 ± 2	3 ± 1	3 ± 1	4 ± 1
BDQ-AMK-RFB-CLR-MTR	1	2 ± 1	2 ± 1	3 ± 1	4 ± 1	4 ± 1	6 ± 1	7 ± 2	3 ± 1	4 ± 1	5 ± 1	6 ± 1	6 ± 1	7 ± 2	7 ± 3	>50
BDQ-AMK-RFB-CLR-MTR-CLS 25	4 ± 1	4 ± 1	4 ± 2	13 ± 1	13 ± 5	28 ± 5	28 ± 4	>50	3 ± 1	4 ± 1	5 ± 1	5 ± 1	5 ± 1	6 ± 1	7 ± 3	7 ± 2
BDQ-AMK-RFB-CLR-MTR-CLS 100	2 ± 1	3 ± 1	4 ± 1	27 ± 4	27 ± 4	>50	>50	>50	5 ± 1	5 ± 2	6 ± 1	7 ± 3	7 ± 2	7 ± 2	19 ± 4	>50

## Data Availability

Not applicable.

## Supplementary Materials

The following supporting information can be downloaded at: https://www.mdpi.com/article/10.3390/microorganisms10071421/s1, Table S1: MICs of Mab-10. Table S2: drugs tested.
