# Supplementary material for: Activity of Drug Combinations against Mycobacterium abscessus Grown in Aerobic and Hypoxic Conditions
Source: Microorganisms. 2022 Jul 14;10(7):1421. doi: 10.3390/microorganisms10071421 (PMC9316547; doi:10.3390/microorganisms10071421)
Supplement: Supplementary file 1 [file microorganisms-10-01421-s001.zip › Supplementary Table S1.pdf]

Supplementary Table S1: MICs of Mab-10

Drug	MAB-10	MIC interpretation (S, I, R) [a]
Clarithromycin	0.25/0.5 [b]	S
Tigecycline	0.25	n.a.
Linezolid	16	I
Amikacin	8 [c]	S
Moxifloxacin	4	R
Ciprofloxacin	2	I
Minocycline	>8	n.a.
Trimethoprim / sulfamethoxazole	4.0/76	R
Tobramycin	8	R
Doxycicline	>16	R
Cefepime	>32	n.a.
Imipenem	16	I
Amoxicillin /clavulanic acid	>64/32	n.a.
Ceftriaxone	>64	n.a.
Cefoxitin	32	I
Bedaquiline	0.125	n.a.
Rifabutin	2	n.a.
Clofazimine	0.06	n.a.

[a] MIC breakpoints from the Clinical and Laboratory Standards Institute (CLSI) [32]: S, susceptible; I, intermediate; R, resistant

[b]Resistance of Mab-10 to clarithromycin was not induced since MIC increased only from 0.25 to 0.5 ug/ml when readings were done on day 5 and 14, respectively. Clarithromycin resistance is due to induction of *erm*(41) gene, and mutations of *rrl* (23S) gene [5]. Whole genome sequencing (WGS) analysis showed that *erm(41)* was not induced because it showed a mutation (Trp10Arg - nucleotide t28c), and that *rrl* had no mutations. Both observations are in keeping with clarithromycin susceptibility assessed by the MICs.

[c]The WGS analysis showed no mutations in the *rrs* (16S) gene, which is involved in amikacin resistance [5]. This observation is in keeping with amikacin susceptibility assessed by the MIC.

n.a., not applicable
