# Supplementary material for: Activity of Drug Combinations against Mycobacterium abscessus Grown in Aerobic and Hypoxic Conditions
Source: Microorganisms. 2022 Jul 14;10(7):1421. doi: 10.3390/microorganisms10071421 (PMC9316547; doi:10.3390/microorganisms10071421)
Supplement: Supplementary file 1 [file microorganisms-10-01421-s001.zip › Supplementary Table S2.pdf]

Supplementary Table S2: drugs tested

Drug (abbreviation)	Class	Major indication	C_{max} (µg/ml)[a] [ref]
Bedaquiline (BDQ)	Dyarilquinoline	Tuberculosis	1 [22]
Amikacin (AMK)	Aminoglycoside	Gram (-) bacteria	8 [21]
Rifabutin (RFB)	Rifamycin	*M. avium* complex	3 [36]
Clarithromycin (CLR)	Macrolide	Gram (+/-) bacteria	2 [37]
Moxifloxacin (MXF)	Fluoroquinolone	Gram (+/-) bacteria	4 [21]
Clofazimine (CLF)	Riminophenazine	Leprosy	1 [22]
Tigecycline (TGC)	Tetracycline	Gram (+/-) bacteria	1 [38]
Linezolid (LNZ)	Oxazolidinone	Gram (+) bacteria	8 [39]
Metronidazole (MTR)	5-nitroimidazole	Anaerobic bacteria & protozoa	8 [21]
Secnidazole (SCN)	5-nitroimidazole	Anaerobic bacteria & protozoa	40 [40]
Tinidazole (TND)	5-nitroimidazole	Anaerobic bacteria & protozoa	40 [41]
Ornidazole (ORN)	5-nitroimidazole	Protozoa infections	10 [42]
Nimorazole (NMR)	5-nitroimidazole	Protozoa. Radiosensitizer	40 [43]
Benznidazole (BNZ)	2-nitroimidazole	Trypanosomiasis	15 [44]
Niclosamide (NCL)	Nitro-benzamide	Tapeworm infections	0.6 [45]
Nitazoxanide (NTZ)	Thiazolide	Anaerobic bacteria & protozoa	10 [25]
Nitroxoline (NTR)	Quinoline derivative	Urinary tract infections	6 [46]
Colistin (CLS)	Lipopeptide	Gram (-) bacteria	25 &100 [47]

[a] Cmax, maximum drug concentration in serum. Cmax in lungs was used for RFB. Concentrations in sputum were used for CLS.
